# Physical Exercise with Music Reduces Gray and White Matter Loss in the Frontal Cortex of Elderly People: The Mihama-Kiho Scan Project

**DOI:** 10.3389/fnagi.2017.00174

**Published:** 2017-06-07

**Authors:** Ken-ichi Tabei, Masayuki Satoh, Jun-ichi Ogawa, Tomoko Tokita, Noriko Nakaguchi, Koji Nakao, Hirotaka Kida, Hidekazu Tomimoto

**Affiliations:** ^1^Department of Dementia Prevention and Therapeutics, Graduate School of Medicine, Mie UniversityTsu, Japan; ^2^Department of Neurology, Graduate School of Medicine, Mie UniversityTsu, Japan; ^3^YAMAHA Music FoundationTokyo, Japan; ^4^Department of Health and Welfare, Mihama Town HallMihama, Japan; ^5^Department of Health and Welfare, Kiho Town HallKiho, Japan; ^6^Department of Neurosurgery, Kinan HospitalTanabe, Japan

**Keywords:** cognition, dementia, human aging, magnetic resonance imaging, voxel-based morphometry

## Abstract

Findings from previous studies suggest that physical exercise combined with cognitive training produces more positive effects on cognitive function in elderly people than physical exercise alone. However, the brain plasticity associated with these proposed benefits of combined therapy has not yet been investigated in elderly subjects. We hypothesized that the dual task group would experience greater benefits than the physical exercise alone and non-exercise control groups with regard to both cognitive function and brain plasticity. This study investigated the effect of physical exercise with musical accompaniment on structural brain changes in healthy elderly people. Fifty-one participants performed physical exercise (once a week for an hour with professional trainers) with musical accompaniment (ExM), 61 participants performed the same exercise without music (Ex), and 32 participants made up the non-exercise group (Cont). After the 1-year intervention, visuospatial functioning of the ExM but not the Ex group was significantly better than that of the Cont group. Voxel-based morphometry analyses revealed that the ExM group showed greater right superior frontal gyrus volume and preserved volumes of the right anterior cingulate gyrus, left superior temporal gyrus, and insula. These results indicate that compared with exercise alone, physical exercise with music induces greater positive effects on cognitive function and leads to subtle neuroanatomical changes in the brains of elderly people. Therefore, physical exercise with music may be a beneficial intervention to delay age-related cognitive decline.

## Introduction

Non-pharmaceutical intervention, especially physical exercise, is associated with positive effects on both cardiovascular fitness and cognitive function of elderly people. Physical activity positively affects cognitive functions such as executive functions, attention, and psychomotor speed (Angevaren et al., 2008; van Uffelen et al., 2008; Smith et al., 2010).

Findings from four previous studies suggest that physical exercise combined with cognitive training produced more positive effects on cognitive function in elderly people than physical exercise alone (Fabre et al., 2002; Oswald et al., 2006; Shatil, 2013; Satoh et al., 2014). For example, we reported that physical exercise combined with music produced more positive effects on cognitive function in elderly people than exercise alone (Satoh et al., 2014). In that study, the physical exercise with musical accompaniment group showed significant improvement in visuospatial function compared to the same exercise without musical accompaniment group and a non-exercise control group. We attributed this improvement to the multifaceted nature of combining physical exercise with cognitive training. However, these previous studies did not unravel the associated neuroanatomical changes in the brain.

Music is one cognitive intervention that is suited for combination with physical exercise in elderly people, and for several reasons. First, listening to music activates a widespread bilateral network of brain regions related to attention, semantic processing, memory, motor functions, and emotional processing (Särkämö et al., 2008, 2014; Bradt et al., 2010). Second, previous studies have reported that listening to music can enhance cognitive function in elderly people (Thompson et al., 2005; Peck et al., 2016). Related to this, music therapy is considered to be grade C1 in the clinical practice guidelines for dementia, with a “recommendation to be done as prevention of cognitive decline in elderly adults, although there is no high level of evidence” (Joint Commission on Guideline for dementing disorder, 2010). Finally, musical rhythm influences physical movement, as demonstrated by improved gait and stride length in patients with Parkinson’s disease who took part in music therapy (McIntosh et al., 1997; Satoh and Kuzuhara, 2008; Schiavio and Altenmüller, 2015).

It is unknown whether physical exercise combined with cognitive training has greater beneficial effects on brain structures in elderly people compared to each intervention alone. The effects of physical exercise on brain tissue loss in the elderly have been extensively studied, and the results suggest that physical exercise positively influences the plasticity of the aging brain (Colcombe et al., 2006; Erickson et al., 2011; Ruscheweyh et al., 2011; Chapman et al., 2013; Niemann et al., 2014). Specifically, participation in an aerobic exercise program increased gray matter volume (GMV) in the prefrontal cortices (Colcombe et al., 2006; Tamura et al., 2015). Aerobic exercise increases the size of the hippocampus (Chapman et al., 2013; Niemann et al., 2014), which has been associated with improved spatial memory (Erickson et al., 2011). However, previous studies showed that physical exercise alone could not preserve parietal region volumes (Erickson et al., 2014). In order to unravel whether a dual task would have greater benefits on cognitive training and brain plasticity than physical exercise alone, it is important to investigate the brain changes related to physical exercise combined with cognitive training.

This investigation was designed to identify structural brain changes related to physical exercise in combination with musical accompaniment in healthy elderly people in the towns of Mihama and Kiho, Mie, Japan. These towns suffer from depopulation, and 35% of the inhabitants are over 65 years old (the mean rate in Japan is 23%). It has been suggested that, considering the aging population, these towns represent the population that will exist throughout Japan in 20 years; therefore, preventing dementia is an urgent problem in the general healthcare project. In the present study, healthy elderly subjects participated in a physical exercise program with or without music accompaniment once a week for 1 year. Neuropsychological assessments and brain magnetic resonance imaging (MRI) were performed before and after the intervention, and the results of the two experimental groups were compared to a control group, which received no intervention. While our previous study found convincing results (Satoh et al., 2014), it did not simultaneously show brain correlates of improved cognitive function. For the first time, we report results of voxel-based morphometry (VBM) from a cohort of participants twice the size of our previous study population. We hypothesized that the dual task group would experience greater benefits than the physical exercise alone and non-exercise control groups with regard to cognitive function and brain plasticity.

## Materials and Methods

### Participants

Participants were recruited from the “Mihama-Kiho Project” in the towns of Mihama and Kiho, Mie, Japan. The project was designed to investigate the effect of physical exercise with and without music on cognitive function and was also a general healthcare project of the towns. The research ethics committee of Kinan hospital approved the experimental protocol, and all participants gave written informed consent prior to the experiment. The study was performed according to the Declaration of Helsinki. This study was registered with the University Hospital Medical Information Network Clinical Trials Registry (UMIN000012148).

To recruit participants, public servants distributed paper flyers among inhabitants 65 years and older who lived in areas of Mihama and Kiho town. The inclusion criteria were as follows: (a) over 65 years old; (b) in good physical and psychological health; (c) normal or corrected vision; (d) ability to clearly hear instructions; (e) living independently; and (f) able to attend an exercise session once a week. Participants were excluded if they met any of the following exclusion criteria: (a) apparent history of cerebrovascular attack; (b) presence of chronic exhausting disease such as malignancy or infection; (c) severe cardiac, respiratory, or orthopedic disability that would prevent participants from exercising; (d) medication that might adversely affect cognition (antidepressants or antipsychotics); or (e) a diagnosis of dementia. The inclusion and exclusion criteria for the control (Cont) group were identical to those for the physical exercise with musical accompaniment (ExM) and the same exercise without musical accompaniment (Ex) groups except for (f) able to attend an exercise session once a week. Instead, participants in the control group were simply required to undergo an MRI and neuropsychological and physiological assessments once a year. Due to budget limitations of the towns, there was no music alone group, and the Cont group recruited was half the size of the intervention groups.

**FIGURE 1:**
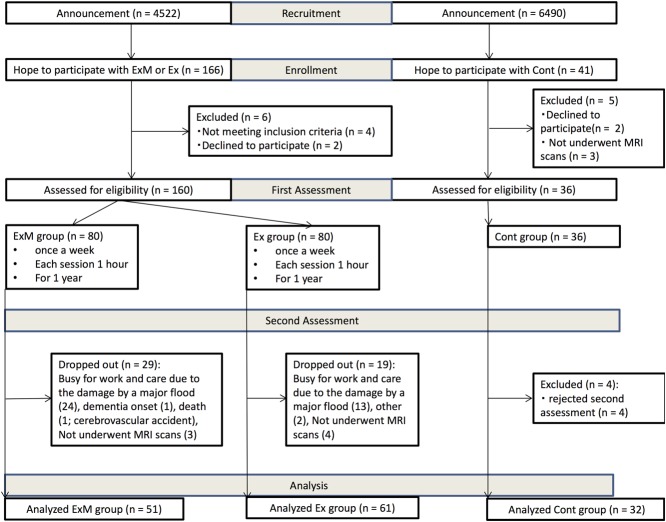
Recruitment flow chart. Cont, control group; Ex, physical exercise without music group; ExM, physical exercise with music group.

Between July 1 and 15, 2011, 166 participants expressed interest in the physical exercise with and without music groups (ExM and Ex, respectively), and 41 participants were interested to be included in the control (Cont) group. Before the intervention, the towns’ public health nurses saw participants and interviewed their family about the subjects’ daily physical and psychological activities. Six participants were excluded because they did not meet the inclusion criteria and declined to participate in the intervention groups. Five participants were excluded from the Cont group because they declined to participate and did not undergo MRI scans. According to age, sex, and activities of daily living (ADL) grade established by the Ministry of Health, Labor and Welfare, the participants were semi-randomly classified into ExM and Ex groups (**Figure 1**). However, married couples and siblings who wished to exercise together were placed in the same group.

No participants switched group. In the ExM group, 29 participants dropped out [busy with work and due to damage by a major flood (*n* = 24), dementia onset (*n* = 1), death (*n* = 1), and did not complete MRI scans (*n* = 3)]. In the Ex group, 19 participants dropped out [busy with work and due to damage by a major flood (*n* = 13), other (*n* = 2), and did not complete MRI scans (*n* = 4)]. In the second assessment, four participants were excluded in the Cont group [rejected second assessment (*n* = 4)]. We analyzed participants whose attendance rate was above 75%. As a result, data from a total of 144 participants were included (**Figure 1**).

### Exercise Intervention

The physical exercise program is described in detail in our previous paper (Satoh et al., 2014). The intervention period was 1 year, and the total number of exercise sessions was 40. Exercise intensity gradually increased with each session. The exercise program and musical accompaniment were developed approximately 10 years ago in collaboration between the Japan Fitness Association, sport medicine experts, and the YAMAHA Music Foundation. The musical accompaniment was synthesizer-heavy dance pop music. The exercise program consists of nine stages, and participants easily and happily performed the exercise. The physical exercise regimen was identical for the ExM and Ex groups and was implemented by professional trainers. To control auditory cue and feedback between the ExM and Ex groups, the ExM group heard music over speakers, while the Ex group only heard a percussive sound that counted the beat over speakers.

### Neuropsychological and Physiological Assessment

Neuropsychological assessment procedures were the same as previously described (Satoh et al., 2014). The Mini Mental State Examination (MMSE) (Folstein et al., 1975) and Raven’s Colored Progressive Matrices (RCPM) (Raven, 1947) were used to screen cognitive ability and quantify intellectual function, respectively. Memory was evaluated using the Logical Memory (LM)-I/-II subtests of the Rivermead Behavioural Memory Test (RBMT) (Wilson et al., 1985), which require immediate and delayed recall of a short story. The RBMT contains four stories with difference levels of difficulty and numbers of words. We used different stories for the pre-and post-testing periods to avoid familiarity with the story content. Visuospatial constructional ability was based on the method described by Strub and Black (2001). Five kinds of figures (vertical diamond, two-dimensional cross, three-dimensional block, three-dimensional pipe, and triangle within a triangle) were shown to the subjects, and they were asked to draw them one by one. Each drawing was scored by assigning one of four possible grades (0: poor, 1: fair, 2: good, and 3: excellent), with a maximum score of 15. Frontal lobe function was assessed using two tasks: word fluency (WF) and Trail-Making Test (TMT)-A/-B (Partington and Leiter, 1949). The WF test consisted of category and letter domains. For the categorical WF, participants were asked to name as many animals as possible in 1 min. For the letter WF task, participants were asked to say the name of objects that begin with each of four phonemes, ka, sa, ta, and te (Dohi et al., 1992). We used the average scores of these four phonemes for statistical analyses. The vital capacity as per cent of predicted (%VC) was used as a physiological assessment.

These neuropsychological and physiological assessments were administered before and after the 1-year intervention period to both the ExM and Ex groups. Cont group participants performed these assessments twice with an interval of 1 year.

### Statistical Analyses

Group differences for demographic variables were examined. The data were assessed using ANOVAs for continuous variables, chi-square tests for dichotomous variables, and Kruskal–Wallis tests for non-parametric data. Post-intervention changes in neuropsychological assessment results between the ExM or Ex group and Cont group were examined. These data were assessed using Dunnett’s tests for continuous variables and the Steele test for non-parametric data. Statistical analyses were done using IBM SPSS Statistics software version 20 (IBM Corp., Armonk, NY, United States) and EZR software version 1.29 (Kanda, 2013).

### MRI Acquisition

All MRI scans were performed with a 1.5-T MRI scanner (Intera, Royal Philips, Netherlands; ECHELON, Hitachi Medical Corporation, Japan). A T1-weighted gradient echo sequence was used (repetition time [TR] = Shortest [Automatic]; echo time [TE] = 15 [Intera]/11 [ECHELON] ms; flip angle = 90°; field of view = 230 mm × 230 mm; slice thickness = 5 mm; in-plane resolution = 0.45 mm × 0.45 mm). The first scans were taken as a baseline before the intervention, and the second scans were taken approximately 1 year later (average interval between pre- and post-intervention: 422.3 ± 34.6 days).

### MRI Analysis

Magnetic resonance imaging data were analyzed using SPM12 (Wellcome Institute of Neurology, University College London, United Kingdom), running on MATLAB R2012a (MathWorks, Natick, MA, United States). In the pre-processing phase, images were set to match the anterior to posterior commissure (AC-PC) line using an automated MATLAB script. Then, images were visually inspected to check for possible scan issues such as field distortion and movement artifacts. Reoriented images were corrected for the intensity inhomogeneity and segmented into gray matter (GM), white matter (WM), cerebrospinal fluid, and other tissues outside the brain by the SPM12 tissue probability maps. The images were registered to the East Asian brains ICBM (International Consortium for Brain Mapping) space template through affine regularization. We created a population-specific template using the SPM12 DARTEL template procedure to directly compare ExM, Ex, and Cont groups and thus investigate (1) GM and WM differences between groups and (2) the relationship between neuropsychological assessment results and GM in these groups at a whole-brain level. The GM and WM segments were inputted into high-dimensional DARTEL to create non-linear modulated-normalized GM and WM images that were smoothed using a Gaussian kernel of 8 mm FWHM (full width at half maximum). No participants were excluded from the analysis after these steps.

For whole-brain and multiple regression analyses, we assessed the statistical significance at a cluster threshold of *p* < 0.05 (family wise error corrected) with a voxel threshold of *p* < 0.001 (uncorrected), and contiguous clusters of at least 10 voxels were reported. We obtained both MNI and Talairach coordinates to detect the anatomical regions of the clusters. We used a transform from Matthew Brett^1^ to convert MNI coordinates to Talairach coordinates, and Talairach Client 2.4.3 (Lancaster et al., 2000) was used to identify the anatomical regions corresponding to Talairach coordinates.

## Results

### Demographics

Participant demographics are shown in **Tables 1A,B**. Although there were no significant differences with regard to the male:female ratio, age, years of education, ADL, MMSE score, or GMV, significant between-group differences were found in white matter volume (WMV) before the intervention (*p* = 0.001). Multiple comparisons revealed that the WMV of the Cont group was larger than that of the ExM and Ex groups (both *p* = 0.001).

**Table 1A:** Baseline demographic information.

	Number (M:F)	Age in years (±*SD*)	Education in years (±*SD*)	ADL-grade (±*SD*)	MMSE score (±*SD*)
ExM	51 (3:48)	71.4 (4.3)	10.8 (2.0)	1.84 (0.46)	27.7 (2.1)
ExM (Drop out)	29 (9:20)	72.1 (5.9)	11.7 (2.5)	1.89 (0.42)	27.21 (2.0)
Ex	61 (8:53)	71.5 (4.6)	11.1 (1.9)	1.80 (0.54)	27.8 (2.0)
Ex (Drop out)	19 (2:17)	74.2 (4.9)	10.1 (2.3)	1.83 (0.71)	27.3 (2.6)
Cont	32 (7:25)	73.6 (5.8)	10.4 (2.2)	1.72 (0.58)	26.8 (2.6)
Cont (Drop out)	4 (1:3)	73.0 (6.5)	9.8 (1.5)	1.5 (0.58)	25.8 (0.5)
*p*-value	0.053^A^	0.13^B^	0.09^C^	0.60^C^	0.21^C^

**Table 1B:** Total GMV and WMV at pre- and post-intervention.

	GMV Pre	GMV Post	WMV Pre	WMV Post
ExM	543.5 (72.7)	538.4 (68.0)	476.3 (79.9)	489.8 (76.7)
Ex	541.0 (55.9)	534.2 (53.2)	466.4 (73.7)	483.2 (70.0)
Cont	515.1 (63.6)	512.1 (72.8)	537.1 (77.8)	541.8 (75.5)
*p*-value	0.117^B^	0.169^B^	0.001^B^	0.001^C^

*ADL, activities of daily life; Cont, control group; Ex, physical exercise without music group; ExM, physical exercise with music group; F, female; GMV, gray matter volume; M, male; MMSE, Mini Mental State Examination; WMV, white matter volume. ^A^ Chi-square test, ^B^ One-way ANOVA, ^C^ Kruskal–Wallis test.*

### Neuropsychological and Physiological Assessment

The neuropsychological and physiological assessment results before and after the interventions are shown in **Table 2**. Intra-group comparisons showed significant improvements in the MMSE score, LM-I and -II subtests of the RBMT, visuospatial assessments, and %VC in both the ExM and Ex groups post-intervention. In addition, significant improvement in the WF category was observed in the Ex group post-intervention. In the Cont group, LM-II and TMT-B scores were significantly improved after 1 year.

**Table 2:** Neuropsychological and physiological assessment results before and after intervention.

Test		Pre- and post-intervention differences, mean (±*SD*)				*p*-value
			ExM	Ex	Cont	
Intelligence	MMSE	Score	1.10^∗3^ (2.1)	0.67^∗1^ (2.3)	0.34 (2.7)	0.34^B^
	RCPM	Score	0.88 (3.6)	0.92 (3.7)	0.00 (3.5)	0.56^B^
		Time	-17.10 (84.3)	8.97 (84.5)	-23.38 (89.5)	0.19^B^
Memory	LM-I		1.20^∗1^ (3.3)	1.13^∗3^ (3.3)	0.31 (4.1)	0.49^A^
	LM-II		1.67^∗3^ (3.4)	1.39^∗2^ (3.5)	1.81^∗2^ (3.3)	0.84^A^
Visuospatial	Copy		1.18^∗4^ (1.7)	0.52^∗3^ (1.4)	0.22 (1.5)	0.038^B^
Frontal	WF	Category	0.45 (4.3)	1.67^∗3^ (4.0)	1.55 (5.5)	0.21^A^
		Letters	0.86 (3.6)	0.98 (4.4)	-0.06 (3.0)	0.43^B^
	TMT	-A	1.04 (31.9)	1.08 (35.5)	-10.31 (50.8)	0.65^B^
		-B	-4.86 (39.3)	-1.47 (55.1)	-37.74^∗1^ (101.8)	0.08^B^
Vital capacity			2.71^∗1^ (8.1)	3.69^∗2^ (11.6)	-2.06 (9.1)	0.031^B^

*Cont, control group; Ex, physical exercise without music group; ExM, physical exercise with music group; LM, Logical Memory of the Rivermead Behavioral Memory Test; MMSE, Mini Mental State Examination; RCPM, Japanese Raven’s Colored Progressive Matrices; SD, standard deviation; TMT, Trail-Making Test; WF, word fluency. Asterisks indicate significant improvement compared with pre-intervention results. ^∗1^p < 0.05, ^∗2^p < 0.01, ^∗3^p < 0.005, ^∗4^p < 0.001. ^A^One-way ANOVA, ^B^Kruskal–Wallis test.*

Significant between-group differences were found for visuospatial assessment and %VC (*p* = 0.038 and 0.031). Multiple comparisons revealed significant differences between the ExM and Cont groups for visuospatial assessment (*p* = 0.037) and between the Ex and Cont groups for %VC (*p* = 0.036).

### GMV

**FIGURE 2:**
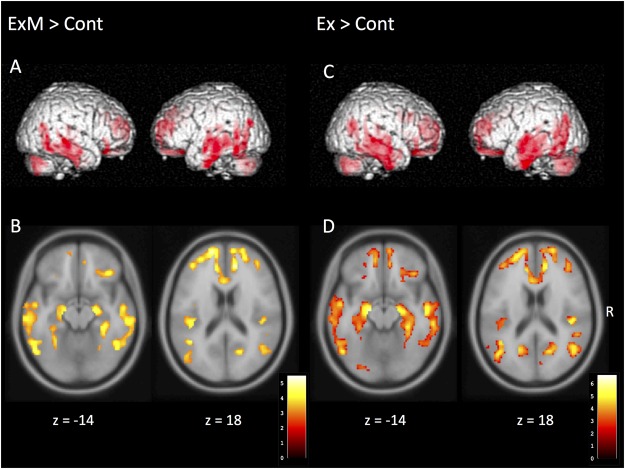
Regions showing a significant post-intervention increase in gray matter volume in the ExM, Ex, and Cont groups. **(A,B)** Gray matter volume was larger in the ExM group relative to the Cont group. **(C,D)** Gray matter volume was larger in the Ex group relative to the Cont group. Cont, control group; Ex, physical exercise without music group; ExM, physical exercise with music group.

Although there were no significant between-group differences in post-intervention GMVs, the volumes were reduced with a declining trend compared to pre-intervention data in each group (**Table 1B**). The following area volumes were significantly larger (preserved) in the ExM group compared to the Cont group: the frontal gyrus (inferior, superior, and medial), cingulate (anterior and posterior), temporal gyrus (inferior, superior, and transverse), insula, parahippocampal gyrus, hippocampus, uncus, fusiform gyrus, thalamus, amygdala, middle occipital gyrus, and cerebellum (**Figures 2A,B** and **Table 3A**). The following area volumes were significantly larger (preserved) in the Ex group compared to the Cont group: the frontal gyrus (inferior, superior, middle, and medial), cingulate (anterior and posterior), temporal gyrus (inferior, superior, middle, and transverse), insula, parahippocampal gyrus, hippocampus, uncus, thalamus, cuneus, precuneus, and cerebellum (**Figures 2C,D** and **Table 3B**).

**Table 3A:** Cluster sizes, peak locations, and statistical values for regions showing significant post-intervention large in the ExM group compared to the Cont group.

				Talairach coordinates (mm)				
Contrast	L/R	Area	BA	*X*	*Y*	*Z*	*Z*-value	Cluster size in voxels
postExM > postCont	R	Inferior frontal gyrus	47	38	31	-12	4.47	1009
	L	Medial frontal gyrus	10	-16	60	3	4.53	11167
	R	Medial frontal gyrus	10	9	59	19	4.65	
	L	Superior frontal gyrus	10	-16	57	16	5.1	
	R	Superior frontal gyrus	10	21	61	11	4.34	
	L	Anterior cingulate	32	-8	29	26	5.11	
	R	Anterior cingulate	32	12	39	13	5.17	
	L	Cingulate gyrus	31	-12	-27	42	4.78	1838
	R	Cingulate gyrus	31	12	-21	40	3.92	
	L	Uncus	20	-32	-7	-33	4.24	9863
	L	Fusiform gyrus	37	-53	-53	-15	4.43	
	L	Inferior temporal gyrus	20	-48	-15	-30	4.72	
	L	Superior temporal gyrus	22	-42	-49	23	4.56	
	L	Transverse temporal gyrus	41	-40	-29	12	4.53	
	L	Middle occipital gyrus	37	-51	-62	-9	4.68	
	R	Parahippocampal gyrus	36	32	-34	-10	4.49	1681
	R	Posterior cingulate	30	14	-61	14	4.44	
	R	Hippocampus		32	-32	-6	4.43	
	R	Thalamus		27	-32	2	4.65	
	R	Parahippocampal gyrus	28	21	-15	-11	4.69	1036
	R	Amygdala		24	-5	-20	3.94	
	R	Uncus	36	27	-3	-28	3.91	
	R	Insula	41	46	-26	14	4.36	6778
	R	Inferior temporal gyrus	20	61	-27	-16	4.66	
	R	Superior temporal gyrus	22	50	-38	6	4.51	
	R	Fusiform gyrus	37	46	-53	-10	4.35	
	R	Middle occipital gyrus	19	48	-59	-5	4.59	
	L	Hippocampus		-28	-33	-3	3.27	1769
	L	Fusiform gyrus	37	-32	-37	-10	3.51	
	L	Inferior semi-lunar lobule		-34	-68	-37	3.43	
	L	Declive		-32	-61	-19	5.13	
	L	Culmen		-32	-53	-15	4.98	
	L	Tuber		-34	-69	-30	3.8	
	L	Uvula		-12	-76	-34	5.33	843
	L	Declive		-21	-77	-21	3.53	
	R	Inferior semi-lunar lobule		26	-74	-39	4.42	1992
	R	Uvula		36	-73	-23	4.92	
	R	Tuber		33	-74	-27	4.83	

**Table 3B:** Cluster sizes, peak locations, and statistical values for regions showing significant post-intervention large in the Ex group compared to the Cont group.

				Talairach coordinates (mm)				
Contrast	L/R	Area	BA	*X*	*Y*	*Z*	*Z*-value	Cluster size in voxels
postEx > postCont	L	Inferior frontal gyrus	11	-12	36	-20	4.83	18328
	R	Inferior frontal gyrus	11	15	28	-20	4.96	
	R	Medial frontal Gyrus	10	12	47	3	5.59	
	L	Superior frontal gyrus	10	-24	51	10	5.1	
	R	Superior frontal gyrus	10	21	61	11	4.95	
	L	Anterior cingulate	32	-8	27	24	5.23	
	R	Anterior cingulate	32	12	39	13	5.21	
	L	Cingulate gyrus	24	-10	-17	41	5.14	
	L	Posterior cingulate	31	-20	-60	18	4.82	1161
	L	Lingual gyrus	18	-15	-55	5	4.13	
	L	Precuneus	31	-16	-60	29	3.82	
	R	Posterior cingulate	31	15	-61	16	5.01	1447
	R	Precuneus	7	21	-61	27	4.31	
	R	Cuneus	17	8	-77	9	3.37	
	R	Inferior frontal gyrus	9	44	7	31	3.96	721
	R	Middle frontal gyrus	46	40	19	24	3.89	
	L	Uncus	36	-30	-5	-32	6.05	39552
	R	Hippocampus		30	-33	-5	5.12	
	R	Insula	41	45	-25	16	5.27	
	L	Inferior temporal gyrus	20	-46	-16	-29	5.42	
	R	Inferior temporal gyrus	20	42	-11	-33	5.3	
	R	Middle temporal gyrus	21	53	-10	-15	5.46	
	L	Superior temporal gyrus	22	-55	-12	2	5.2	
	R	Superior temporal gyrus	22	51	-18	-8	5.84	
	R	Transverse temporal gyrus	41	46	-26	12	5.35	
	L	Parahippocampal gyrus	28	-18	-14	-12	5.96	
	R	Parahippocampal gyrus	28	21	-15	-11	5.3	
	R	Thalamus		27	-31	2	5.68	
	L	Uvula		-12	-75	-33	6.42	
	L	Declive		-33	-59	-20	5.67	

*BA, Brodmann area; Cont, control group; Ex, physical exercise without music group; ExM, physical exercise with music group; Ex, physical exercise without music group; L, left; R, right.*

**FIGURE 3:**
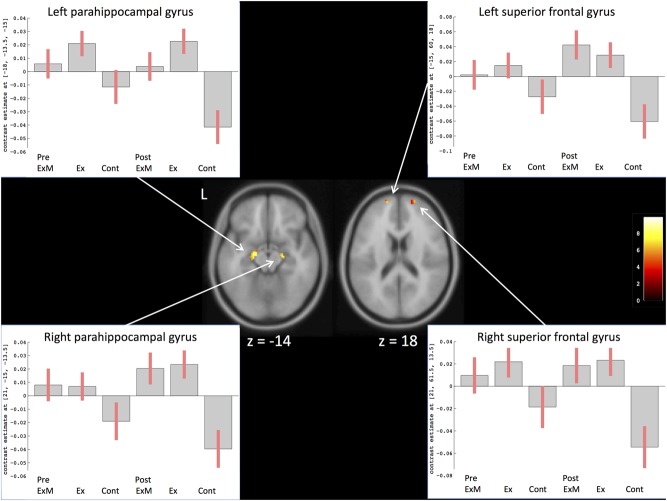
Changes in gray matter volume in different brain areas after intervention. The axis of ordinate displays changes based on β parameter estimates (with 90% confidence intervals) of the peak voxels within the respective clusters. As a region of interest (ROI), we compared pre- and post-intervention contrasts between the three groups to eliminate the possibility of pre-intervention differences in GMV: (post_ExM > pre ExM) > (post_Cont > pre_Cont) and (post_Ex > pre_Ex) > (post_ Cont > pre_Cont).

To compare the amount of GMV change between the ExM or Ex group and Cont group, we contrasted the post- and pre-intervention data for each group: (post_ExM > pre_ExM) > (post_Cont > pre_Cont) and (post_Ex > pre_Ex) > (post_Cont > pre_Cont). These contrasts showed larger volumes of the bilateral superior frontal and parahippocampal gyri in the ExM and Ex groups compared to the Cont group (*p* < 0.05 uncorrected). To distinguish pre- and post-intervention volume differences in different brain areas for each group, we calculated 90% confidence intervals by using these contrasts as a region of interest (ROI) (*p* < 0.001, uncorrected; **Figure 3** and **Table 4**). The volumes of the ExM and Ex groups were preserved and actually increased. The right superior frontal gyrus volume only increased in the ExM group. In the Cont group, these areas were reduced after 1 year.

**Table 4:** Cluster sizes, peak locations, and statistical values for each contrast.

				Talairach coordinates (mm)				
Contrast	L/R	Area	BA	*X*	*Y*	*Z*	*Z*-value	Cluster size in voxels
Effect of differences of pre and post (GM)	L	Hippocampus	28	-18	-14	-12	4.99	84
	R	Hippocampus	28	21	-15	-11	4.61	29
	L	Superior frontal gyrus	10	-15	59	14	4.52	31
	R	Superior frontal gyrus	10	21	61	10	2.5	69
Effect of differences of pre and post (WM)	R	Superior frontal gyrus	6	12	22	52	5.39	29
Regression with visuospatial score	L	Superior temporal gyrus	22	-48	-10	3	4.66	977
	L	Insula	13	-34	-23	14	4.21	
	L	Anterior cingulate	24	-4	25	26	4.6	2384
	R	Anterior cingulate	32	8	36	20	4.18	
	R	Anterior cingulate	32	12	39	13	3.74	

*BA, Brodmann area; GM, gray matter; pre, pre-intervention; post, post-intervention; WM, white matter.*

### WMV

Significant between-group differences were found for post-intervention WMV (*p* = 0.001). Multiple comparisons revealed that the WMV of the Cont group was larger than those of the ExM and Ex groups (*p* = 0.009 and 0.001, respectively).

**FIGURE 4:**
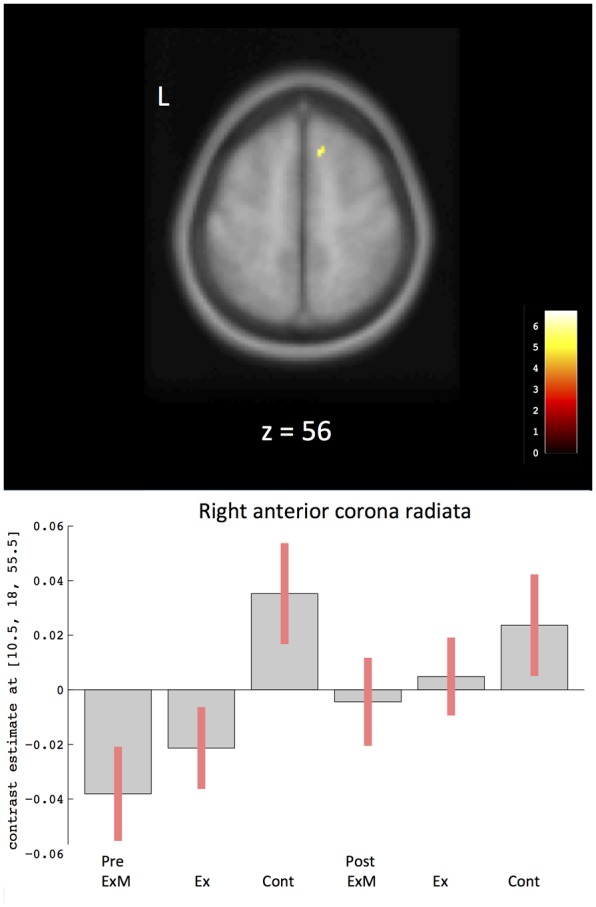
Changes in white matter volume in different brain areas after intervention. The axis of ordinate displays changes based on β parameter estimates (with 90% confidence intervals) of the peak voxels within the respective clusters. As a ROI, we compared pre- and post-intervention contrasts between the three groups to eliminate the possibility of pre-intervention differences in WMV: (post_ExM > pre ExM) > (post_Cont > pre_Cont) and (post_Ex > pre_Ex) > (post_Cont > pre_Cont). Cont, control group; Ex, physical exercise without music group; ExM, physical exercise with music group.

To compare the amount of WMV change between the ExM or Ex group and the Cont group, we contrasted the post-intervention and pre-intervention data for each group: (post_ExM > pre ExM) > (post_Cont > pre_Cont) and (post_Ex > pre_Ex) > (post_ Cont > pre_Cont). These contrasts showed a larger right anterior corona radiata volume in the ExM and Ex groups compared to the Cont group (*p* < 0.001, uncorrected). To determine pre- and post-intervention volume differences, we calculated a 90% confidence interval by using these contrasts as an ROI (**Figure 4** and **Table 4**). WMV was increased in the ExM and Ex groups post-intervention, whereas WMV of the Cont group was reduced compared to baseline.

### Correlation between Neuropsychological Assessments and GMV

**FIGURE 5:**
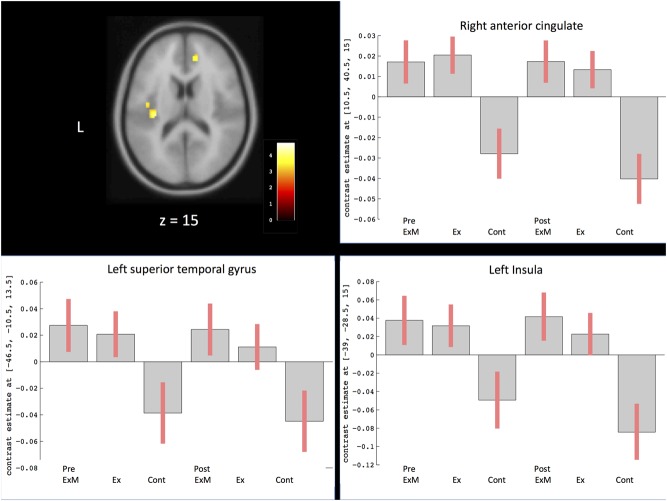
Region showing regional gray matter volume correlated with visuospatial assessment. The axis of ordinate displays changes based on β parameter estimates (with 90% confidence intervals) of the peak voxels within the respective clusters in each group pre- and post-intervention. Cont, control group; Ex, physical exercise without music group; ExM, physical exercise with music group.

Based on the visuospatial assessment results only showing a significant difference between the ExM and Cont groups, we found that changes in visuospatial scores were positively correlated with the volumes of the left superior temporal gyrus, right anterior cingulate gyrus, and left insula (**Figure 5** and **Table 4**). We calculated a 90% confidence interval by using these contrasts as an ROI. Although the volumes of the ExM group were preserved, those of the Ex and Cont groups were reduced (**Figure 5**).

## Discussion

This investigation was designed to identify structural brain changes related to physical exercise in combination with musical accompaniment in healthy elderly people. The present study demonstrated that physical exercise in combination with music can produce greater benefits than physical exercise alone, for both cognitive function and brain plasticity in elderly subjects. Although the content and duration of exercise were identical for the ExM and Ex groups, only the ExM group showed significant improvement in visuospatial function compared to the Cont group. These results are consistent with those of our previous study (Satoh et al., 2014), which had a sample size half that of the present study. Furthermore, for the first time we report VBM results; these showed that only the ExM group showed an increase in right superior frontal gyrus volume and preserved volumes for the left superior temporal gyrus, right anterior cingulate gyrus, and left insula. Our results are similar to those reported in mouse models of Alzheimer disease; the studies reported the positive effects of physical training in mouse models of Alzheimer disease (García-Mesa et al., 2011), and showed the advantages of joint physical and social training in adolescent and young adult mice (Madroñal et al., 2010). The authors concluded that benefits of aerobic physical exercise on synapse, redox homeostasis, and general brain function further support the value of a healthy life-style against neurodegeneration.

Regarding the correlation between cognitive function and regional GMV, changes in visuospatial assessment were positively correlated with volume changes of the left superior temporal gyrus, right anterior cingulate gyrus, and left insula. Visuospatial processing consistently activates superior and inferior parietal regions, which underlie spatial attention, and frontal areas such as the dorsolateral prefrontal cortex and anterior cingulate gyrus, which reflect working memory components (Cohen et al., 1996; Silk et al., 2006). Therefore, changes in visuospatial assessment should be preserved along with the frontal and parietal region volumes. However, our results showed that only the anterior cingulate gyrus was preserved in the ExM group, which also showed a significant improvement in visuospatial processing compared to the other groups. Volumes of the parietal regions decreased in all groups, and there were no significant post-intervention between-group differences. Physical exercise with musical accompaniment did not preserve the volume of parietal regions, which is similar to what has been previously reported with physical exercise alone (Colcombe et al., 2006; Erickson et al., 2011, 2014; Ruscheweyh et al., 2011). Therefore, preserved frontal area volumes might explain why visuospatial processing was only improved in the ExM group.

On the contrary, the decreased frontal cortex volume in the Cont group is consistent with an earlier report (Raz et al., 2004); the frontal cortex undergoes age-related changes or declines in both volume and function. As the anterior cingulate gyrus was only preserved in the ExM group, our results imply that music might slow the multi-factorial age-related decline of frontal cortex functions. Music may therefore be an effective intervention to preserve frontal cortex volume.

In addition, both LM-I and -II memory assessment scores significantly improved in ExM and Ex groups after the intervention. The hippocampus plays an important role in memory. We found that the hippocampus and parahippocampal gyrus volumes of the ExM and Ex groups were preserved and therefore, were greater than those measured in the Cont group, which corresponds to these LM-I and -II results. Erickson et al. (2011) reported that aerobic exercise training increases the size of the anterior hippocampus, which is associated with improved spatial memory. Herting and Nagel (2012) found that higher aerobic fitness predicted better learning and larger hippocampal volumes in adolescents. Thus, our results confirm earlier reports that physical exercise positively influences memory.

MMSE scores were also significantly improved in both the ExM and Ex groups. Thus, our results confirm earlier reports that physical exercise positively influences cognitive function (Angevaren et al., 2008; van Uffelen et al., 2008; Smith et al., 2010).

There are some limitations to this study. First, because participants were willing to attend the exercise program provided by their towns, there might have been a selection bias among the ExM and Ex groups. Moreover, randomization was not pure due to sibling and spouse clustering, and this should be addressed in future studies. With the exception of WMV at baseline, brain volumes were not significantly different among the three groups before the intervention. Thus, the innate capacities and regional brain volumes of the participants of the ExM and Ex groups might have been different from those of the Cont group. Second, the intervention period was only 1 year. A longer intervention period may have more pronounced effects and prevent cognitive deterioration and brain volume decreases to a greater degree. Additional investigations will be needed to understand these longitudinal effects of combined training on brain volume. Lastly, it is impossible to eliminate the influence of the learning effect on the neuropsychological assessment results. However, in the pre- and post-intervention LM test, we used different stories that had the same difficulty levels and numbers of words and sentences. In addition, because the present results showed significant differences among the ExM, Ex, and Cont groups after the same time interval, we do not feel that learning effects contributed to our findings. Further research is required to quantify the optimal amount of physical training while listening to music. It will be important to include a control group for which physical exercise is combined with a secondary non-music task to disentangle the specific effects of music on cognitive training.

## Conclusion

Exposure of elderly subjects to physical exercise with music improved visuospatial processing and attenuated age-associated volume decreases in frontal GM and WM. These results suggest that physical exercise with music could delay age-related cognitive decline.

## Author Contributions

Conception and design of the experiments: MS. Conduction of the experiments: TT, NN, and KN. Data analysis: KT and MS. Writing of the paper: KT and MS. Contribution of materials: JO. Analysis and interpretation of the data: HK. Supervision and interpretation of the data: HT.

## Conflict of Interest Statement

The authors declare that the research was conducted in the absence of any commercial or financial relationships that could be construed as a potential conflict of interest.

## Footnotes

1 http://imaging.mrc-cbu.cam.ac.uk/imaging/MniTalairach
